# Bioinformatics analysis of rheumatoid arthritis tissues identifies genes and potential drugs that are expressed specifically

**DOI:** 10.1038/s41598-023-31438-6

**Published:** 2023-03-18

**Authors:** Qingshan He, Hanmeng Ding

**Affiliations:** Nanyang Medical College, Henan, 473000 China

**Keywords:** Biomarkers, Diseases, Rheumatology

## Abstract

Studies have implicated necroptosis mechanisms in orthopaedic-related diseases, since necroptosis is a unique regulatory cell death pattern. However, the role of Necroptosis-related genes in rheumatoid arthritis (RA) has not been well described. We downloaded RA-related data information and Necroptosis-related genes from the Gene Expression Omnibus (GEO), Kyoto Gene and Genome Encyclopedia (KEGG) database, and Genome Enrichment Analysis (GSEA), respectively. We identified 113 genes associated with RA-related necroptosis, which was closely associated with the cytokine-mediated signaling pathway, necroptosis and programmed necrosis. Subsequently, FAS, MAPK8 and TNFSF10 were identified as key genes among 48 Necroptosis-associated differential genes by three machine learning algorithms (LASSO, RF and SVM-RFE), and the key genes had good diagnostic power in distinguishing RA patients from healthy controls. According to functional enrichment analysis, these genes may regulate multiple pathways, such as B-cell receptor signaling, T-cell receptor signaling pathways, chemokine signaling pathways and cytokine-cytokine receptor interactions, and play corresponding roles in RA. Furthermore, we predicted 48 targeted drugs against key genes and 31 chemical structural formulae based on targeted drug prediction. Moreover, key genes were associated with complex regulatory relationships in the ceRNA network. According to CIBERSORT analysis, FAS, MAPK8 and TNFSF10 may be associated with changes in the immune microenvironment of RA patients. Our study developed a diagnostic validity and provided insight to the mechanisms of RA. Further studies will be required to test its diagnostic value for RA before it can be implemented in clinical practice.

## Introduction

The chronic autoimmune disease rheumatoid arthritis (RA) causes limb deformities and limb disability, but there are individual differences in disease and disease progression. There is about 1% prevalence of RA in the adult population, primarily affecting women in their middle to late stages of life^1^. In addition to being the leading cause of disability in the world, RA has a significant impact on public health and the economy^2^. The clinical features of RA disease are characterized by synovial cell infiltration and survival, leading to cartilage damage, synovial thickening, bone erosion and local inflammatory cell infiltration of the joint, resulting in chronic inflammation. The pathogenesis of RA involves the activation of osteoclasts by inflammatory cytokines such as tumor necrosis factor alpha, interleukin 6, and intercellular matrix, leading to the destruction of RA patients and cartilage^3,4^. The main clinical treatment options for the pathogenesis of RA include biological agents, non-steroidal anti-inflammatory drugs and glucocorticoids, and surgical treatment, but there is still a lack of effective therapeutic targets for RA synovium. The search for potential therapeutic targets for RA is therefore of utmost importance.

It was discovered in 1988^5^ and named in 2005 that necroptosis is a novel pattern of regulated cell death^6^. Numerous factors have been found to trigger Necroptosis, including: members of the tumor necrosis factor (TNF) family, and other pathogenic microorganisms^6^. The inhibition of cysteine protease 8 (caspase 8) suppresses apoptosis and activates necroptosis^7^. RIP1 and RIP3 bind to form a complex called "necrosome", which then induces RIP3 autophosphorylation. formation, translocation to the cell membrane, leading to its rupture and eventual destruction of the cell^8^. Recent studies have suggested that necroptosis mechanisms play an important role in the pathogenesis of orthopaedic-related diseases, including osteoarthritis^9^. In osteoarthritic cartilage, the expression of Necroptosis markers RIPK1 and RIPK3 was upregulated after stimulation by inflammatory factors such as tumor necrosis factor ɑ^10^, and inhibition of Necroptosis significantly reduced articular cartilage destruction and delayed the progression of osteoarthritis^11^, effectively confirming that Necroptosis plays an important role in the progression of osteoarthritis. There are few reports on RA and Necroptosis, and its mechanism of action is still unclear. In order to further understand the pathogenesis of RA from the perspective of Necroptosis, it is important to uncover potential targets.

This study used the Gene Expression Omnibus (GEO) database to conduct a systematic analysis of immune infiltrates. Consequently, evaluating the role of Necroptosis in the development of RA. Simultaneously, we investigate the potential functional mechanisms of Necroptosis in RA, the relationship between key genes and infiltrating immune cells to have a better understanding of the underlying molecular immune processes in the progression of RA.

## Materials and methods

### Data download and collation

To conduct this study, we retrieved RA data from the Gene Expression Omnibus (GEO, http://www.ncbi.nlms.nih.gov/geo) using the keyword "rheumatoid arthritis". As training datasets for discovery, the GSE55235 dataset and the GSE55457 dataset were constructed from the GPL96 [HG-U133A] Affymetrix Human Genome U133A Array platform, containing synovial tissue from 23 RA patients and 20 healthy control synovial tissues^12^. Several RA patients' synovial tissue and 16 healthy control synovial tissues were used for independent external validation of the microarray dataset GSE77298, which included synovial tissue from sixteen RA patients and seven healthy controls^13^. GEO provided all of the data for the study, so informed consent and ethical approval were not required.

### Differential expression analysis

First, we collected 159 Necroptosis-associated genes from the Kyoto Gene and Genome Encyclopedia (KEGG) database and Genome Enrichment Analysis (GSEA)^14^. As well as performing differential analysis of Necroptosis-associated genes (NRDEGs), we used R software's "limma"^15^ package to identify significant genes.

### Analysis of functional enrichment

The purpose of this study was to further understand the biological functions and significance of NRDEGs in RA by investigating gene ontology (GO) enrichment analysis and KEGG enrichment analysis. Among them, "ggplot2", "clusterProfiler"^16^, "org.Hs.eg.db" and "enrichplot " packages were used to perform enrichment analysis of NRDEGs, and significantly enriched GO and KEGG terms were screened at corrected *p* value < 0.05.

### Identify the best key genes for the diagnosis of RA

The NRDEGs were summed using three machine learning algorithms (LASSO, SVM-RFE, and RF). The least absolute shrinkage and selection operator (LASSO) and support vector machine (SVM) were used to classify diagnostic markers for RA. In order to distinguished RA patients from healthy controls, a cross-validation of 10 folds was conducted using the "glmnet"^17^ package. The SVM-REF algorithm is able to generate a hyperplane with maximum margin in the feature space to distinguish between positive and negative instances. Here, we use the "e1071" and "svmRadial" packages in R software to perform the SVM-RFE algorithm analysis to screen for quality genes. Recursive partitioning is used to construct binary trees in random forests (RFs)^18^. We used the "randomForest" package to construct an RF classification model to rank Necroptosis-related genes according to the Gini index to screen for characteristically expressed NRDGEs. The crossover genes of the three machine learning algorithms were considered as key genes for the diagnostic analysis of RA. The "pROC"^19^ package was used to evaluate the predictive power of RA diagnosis. The AUC of the ROC was calculated to determine the accuracy of the prediction model; the higher the AUC value, the higher the accuracy of the prediction model.

### Genome enrichment analysis (GSEA)

We used the "c2.cp.kegg.v11.0.symbols" gene set^20^ from Molecular Signature Data Lane (MSigDB, http://software.broadinstitute.org/gsea/msigdb) as a reference set to perform GSEA analysis of key genes to explore their biological significance and function. We set the significance threshold at corrected *p* value < 0.05 and the number of random sample alignments at 1000 to ensure that enrichment scores are normalized in the analysis.

### Immune cell infiltration analysis

CIBERSORT a method for characterizing the cellular composition of complex tissues from gene expression profiles^21^. We used the CIBERSORT algorithm (http://cibersortx.stanford.edu) to determine the relative proportion of each of the 22 types of invading immune cells (Supplementary Table 1). Furthermore, the "Spearman" rank correlation analysis in R software was used to determine the association between signature genes and the number of infiltrating immune cells. The graphical approach of the "ggplot2" software package was used to display the resulting correlations.

### Key gene drug prediction and construction of ceRNA network

We screened key gene-associated targeted drugs by DGIdb (https://dgidb.genome.wustl.edu/) database, and the relevant screening conditions were all default values. Moreover, we analyzed NCBI's mRNA-miRNA interaction data and the StarBase (http://starbase.sysu.edu.cn) database to predict mRNA-miRNA interactions of key genes. Finally, the predicted miRNAs were searched in starBase and screened for miRNA-lncRNAs, resulting in a ceRNA network of mRNA-miRNA-lncRNAs. Among them, Cytoscape (3.7.1) software was used to visualize the analysis.

### Validation of key genes

The expression of NRDEGs was extracted from the GSE77298 dataset, with the software packages "ggpubr" and "ggplot2", we calculated and visualized differences between RA and normal samples. Corrected *p* values less than 0.05 were considered to be significant.

## Results

### Identification of Necroptosis-related differential genes

**Figure 1:**
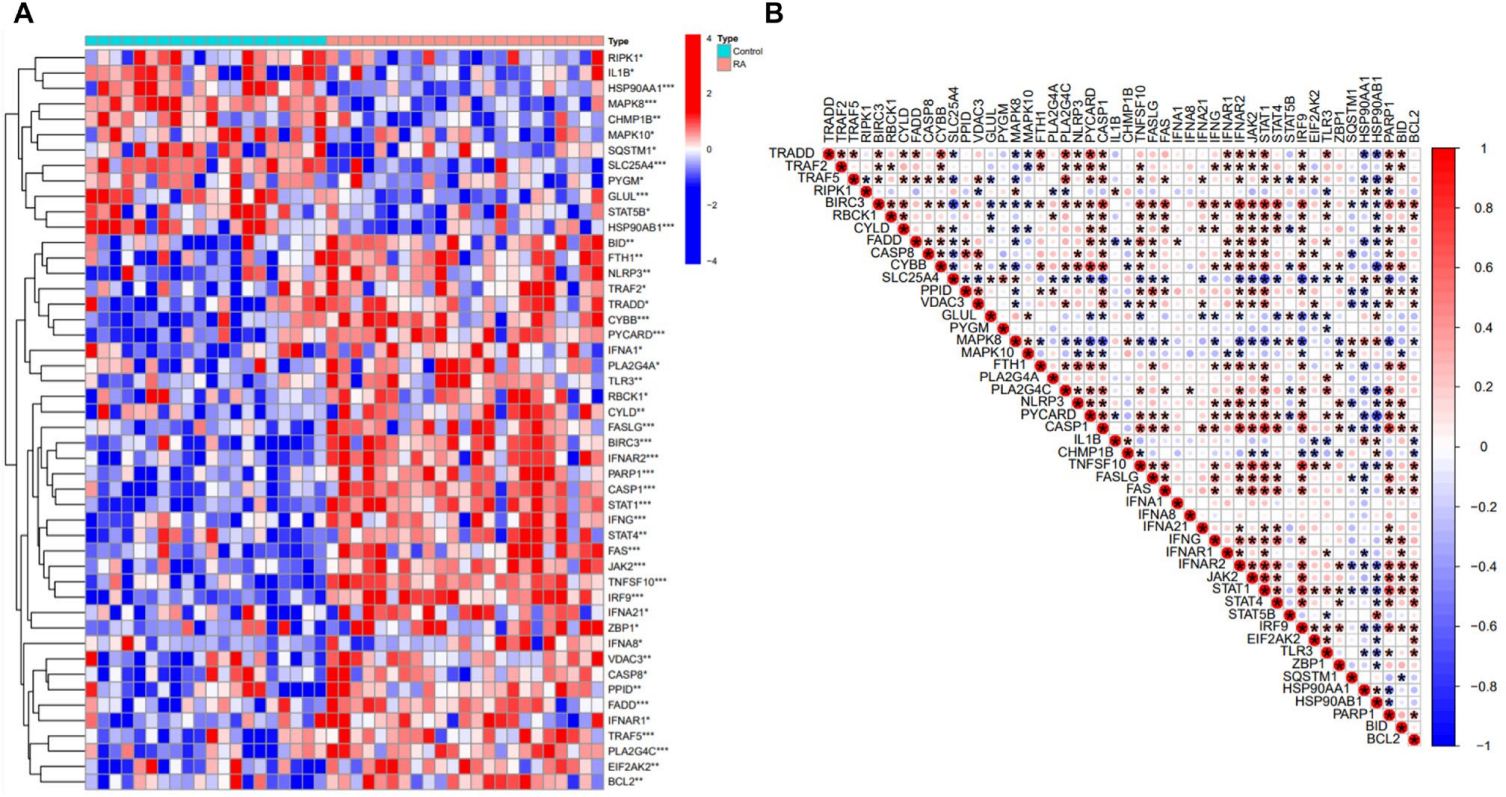
Analysis of expression levels of Necroptosis-related differential genes in RA. (**A**) Heat map showing the expression pattern of NRDEGs in different samples. (**B**) Correlation between the NRDEGs.

In the experimental data set (GSE55235 and GSE55457), 23 RA patient synovial tissues and 20 healthy control synovial tissues were included. The results of the difference analysis showed that a total of 48 out of 113 Necroptosis-related genes were found to be different between RA synovial tissue samples and healthy control samples, including 36 Among them, 36 genes were upregulated and 12 genes were downregulated. The clustering heat map showed the expression pattern of Necroptosis differential genes (NRDEGs) in RA samples and healthy control samples (Fig. 1A), and the correlation between NRDEGs is shown in Fig. 1B. FAS was positively correlated with MAPK8, BIRC, JAK2 and STAT1, and MAPK8 was negatively correlated with PYCARD, CASP1 and TNFSF10. Interestingly, IFNA1 and IFNA8 were positively correlated with FADD and PLA2G4C only.

### Enrichment analysis of NRDEGs

**Figure 2:**
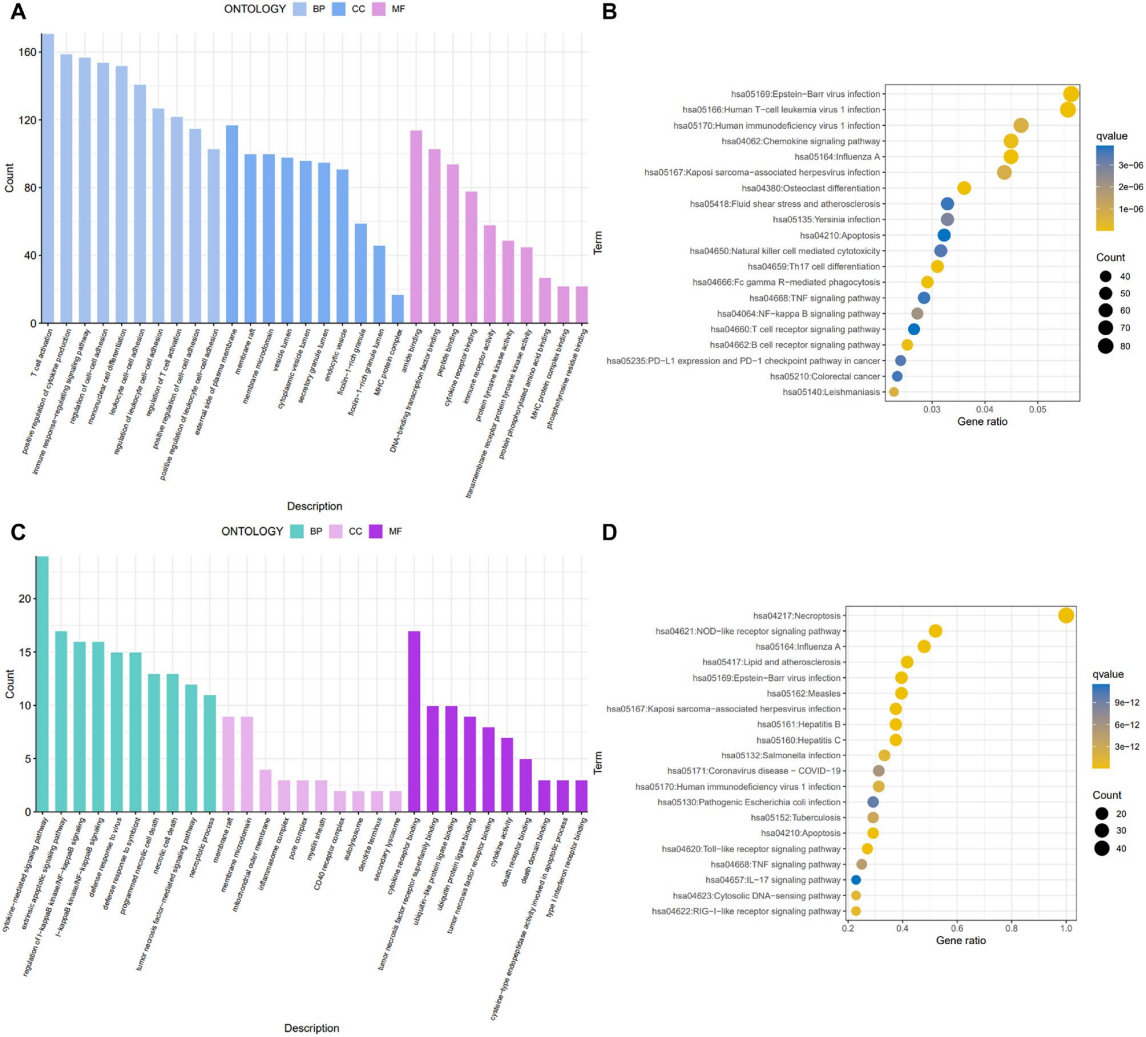
Functional analysis of NRDEGs. (**A**) The GO enrichment analysis results showed that T cell activation, external side of plasma membrane and amide binding were significantly enriched in RA. (**B**) KEGG results showed that "Epstein-Barr virus infection" and "Human T-cell leukemia virus 1 infection" were significantly enriched in RA. (**C**) NRDEGs were closely related to cytokine-mediated signaling pathway, membrane raft and cytokine receptor binding. (**D**) NRDEGs were closely related to pathways such as "Necroptosis" and "NOD-like receptor signaling pathway".

We enriched for RA-differentiated genes and NRDEGs (including GO enrichment analysis and KEGG analysis) to understand the biological functions and related pathways of NRDEGs in RA. The GO enrichment analysis results showed that T cell activation, external side of plasma membrane and amide binding were significantly enriched in RA, while NRDEGs were closely related to cytokine-mediated signaling pathway, membrane raft and cytokine receptor binding. Among them, the molecular function of "membrane raft" was commonly enriched (Fig. 2A,C). In addition, KEGG results showed that "Epstein-Barr virus infection" and "Human T-cell leukemia virus 1 infection" were significantly enriched in RA, while NRDEGs were closely related to pathways such as "Necroptosis" and "NOD-like receptor signaling pathway" (Fig. 2B,D). In summary, the enrichment analysis evidence suggests that NRDEGs may play an important role in the development of RA through the regulation of "cytokine-mediated signaling pathway", "membrane raft", "Necroptosis" and "NOD-like receptor signaling pathway".

### Three genes in NRDEGs were identified as key genes for RA

**Figure 3:**
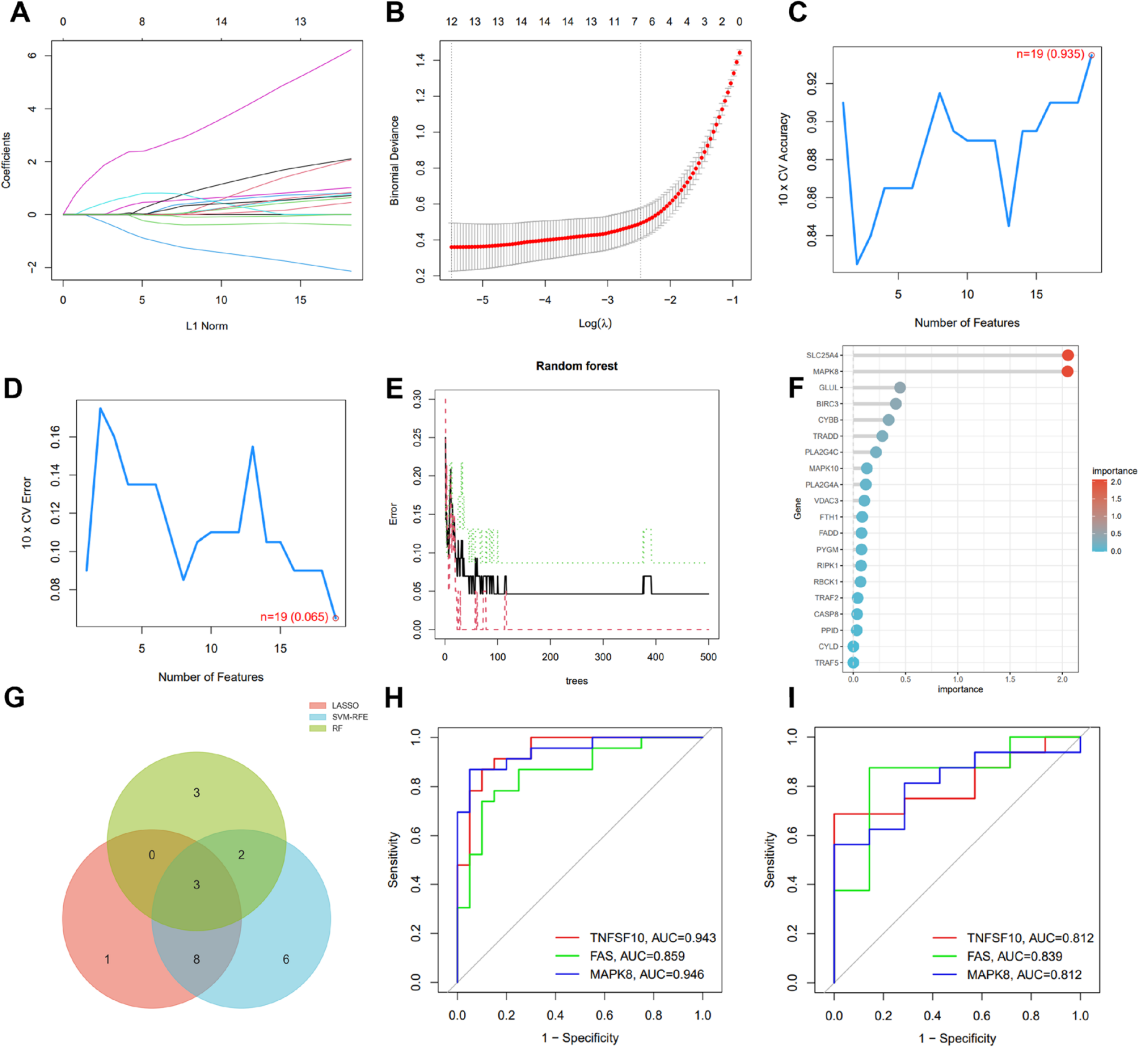
Three genes in NRDEGs were identified as key genes for RA. (**A**–**B**) Penalized parameter adjustment by LASSO logistic regression algorithm with tenfold cross-validation was used to select 12 RA-related features. (**C**–**D**) SVM-RFE algorithm to filter 48 NRDEGs to determine the best combination of key genes. Finally, 19 genes (maximum precision = 0.935, minimum RMSE = 0.065) were identified as the best key genes. (**E**–**F**) Key gene screening was performed by random forest algorithm, and 8 genes were identified as key genes based on gene importance greater than 0.9. (**G**) Key genes obtained from LASSO, SVM-RFE and RF models. (**H**) ROC curves of the 3 key genes in the training set. (**I**) ROC curves of the 3 key genes in the validation set.

In light of the distinct differences between people with RA and healthy individuals, we assessed NRDEGs' diagnostic potential. We screened meaningful key genes in the training dataset by three machine learning algorithms (LASSO, SVM-RFE and RF) for the purpose of differentiating RA patients. In the LASSO algorithm, 12 genes out of 48 NRDEGs were selected (Fig. 3A,B). Meanwhile, in the SVM-RFE algorithm, we identified 19 NRDEGs(maximum precision = 0.935, minimum RMSE = 0.065) (Fig. 3C,D). RF algorithms were used to determine gene importance for NRDEGs, and we identified eight genes with an importance greater than 0.9 bits (Fig. 3E-F). Finally, we combined the results of the three machine learning algorithms, with three genes identified as key genes (FAS, MAPK8 and TNFSF10, Fig. 3G). In order to investigate whether three key genes could be used to differentiate RA samples from healthy controls, we plotted ROC curves. As shown in Fig. 3H, the AUCs of the key genes were all greater than 0.85. In addition, we also plotted the ROC curves of the validation dataset to further validate the diagnostic potential of the key genes. The results showed that the AUC values of the three key genes in the validation dataset were all greater than 0.8, indicating that these three key genes have some accuracy and specificity for differentiating RA samples from normal control samples (Fig. 3I). The above evidence suggests that the key genes have high accuracy and specificity for differentiating RA patients from healthy control samples.

### GSEA analysis of key genes

**Figure 4:**
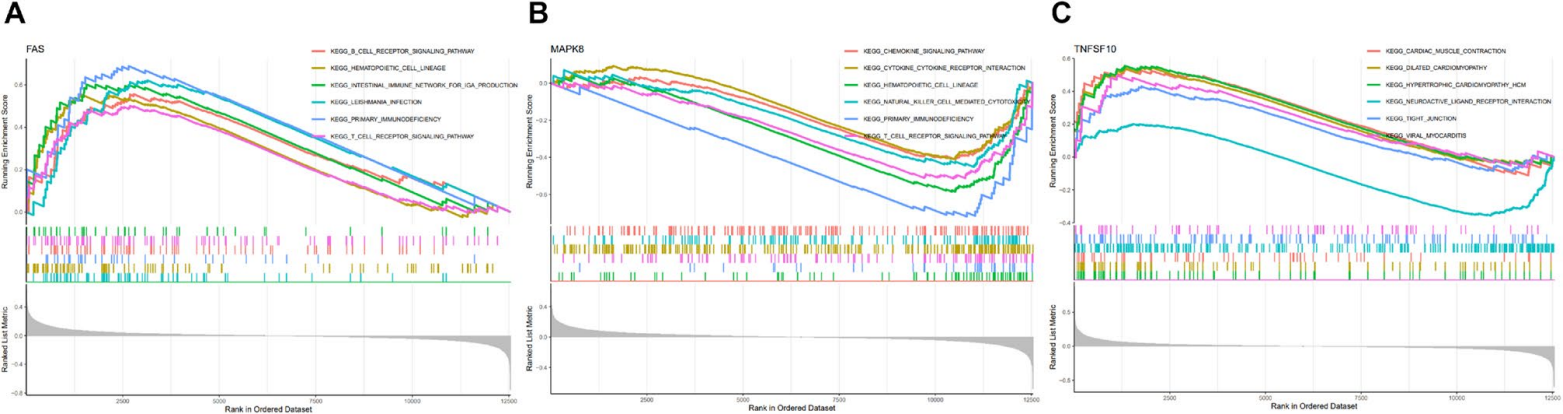
Single-gene GSEA-KEGG pathway analysis of FAS (**A**), MAPK8 (**B**) and TNFSF10 (**C**).

Since the potential function of key genes in RA is unknown to us, GSEA analysis was performed to understand their role and significance in RA. The first six pathways associated with key gene enrichment are shown in Fig. 4. A comprehensive analysis revealed that key genes were mainly involved in immune responses (B-cell receptor signaling pathway, T-cell receptor signaling pathway, chemokine signaling pathway and cytokine-cytokine receptor interaction) and various disease pathways (viral myocarditis, tension arthritis, dilated cardiomyopathy and hypertrophic cardiomyopathy).

### Immune cell infiltration analysis

**Figure 5:**
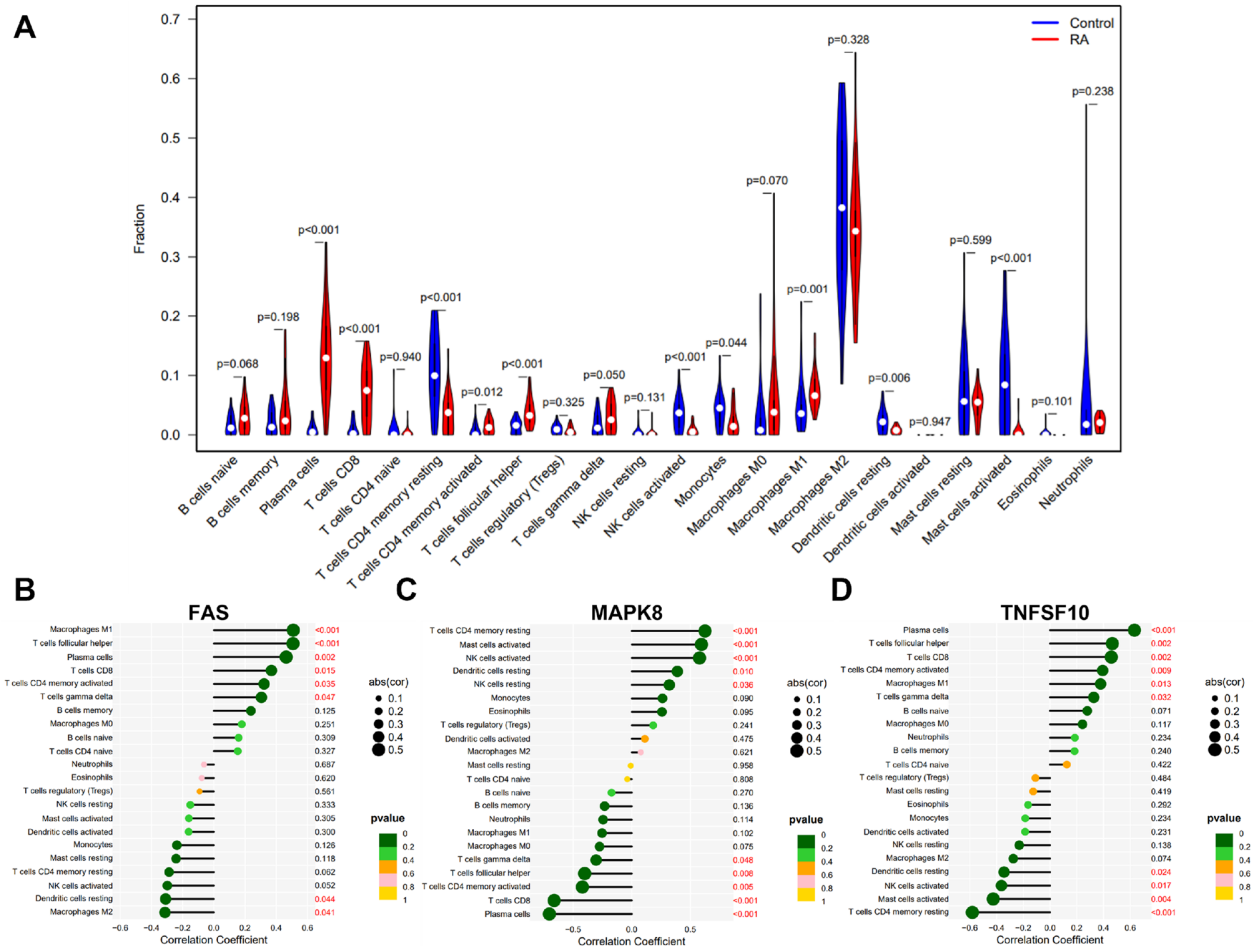
Immune cell infiltration analysis. (**A**) Implementation of the CIBERSORT algorithm to explore the differences in immune microenvironment between RA patients and normal samples. (**B**) Graph of key genes and immune cell correlation analysis.

In addition to being complex in pathogenesis, RA is greatly influenced by the immune system^22,23^. In order to investigate the differences in immune microenvironment between RA patients and healthy controls, we employed the CIBERSORT algorithm. As shown in Fig. 5A, the proportions of Plasma cells, T cells CD8, T cells follicular helper and Macrophages M1 were higher in RA samples than in healthy control samples, while T cells CD4 memory resting, NK cells activated, Monocytes and Mast cells activated were more expressed in the healthy control samples. In addition, we analyzed the relationship between high and low expression of characteristic genes and the immune microenvironment. FAS and MAPK8 were significantly positively correlated with them, whereas TNFSF10 was negatively correlated with them in immune cells T cells follicular helper, Plasma cells, T cells CD8, T cells CD4 memory activated and T cells gamma delta. A significant negative correlation was found between FAS and MAPK8, as well as a positive correlation between TNFSF10 and Dendritic Cells while they were resting (Fig. 5B–D). Based on these evidences, FAS, MAPK8 and TNFSF10 may strongly influence the immune microenvironment of RA patients.

### Prediction of key gene-targeted drugs and construction of ceRNA networks

**Figure 6:**
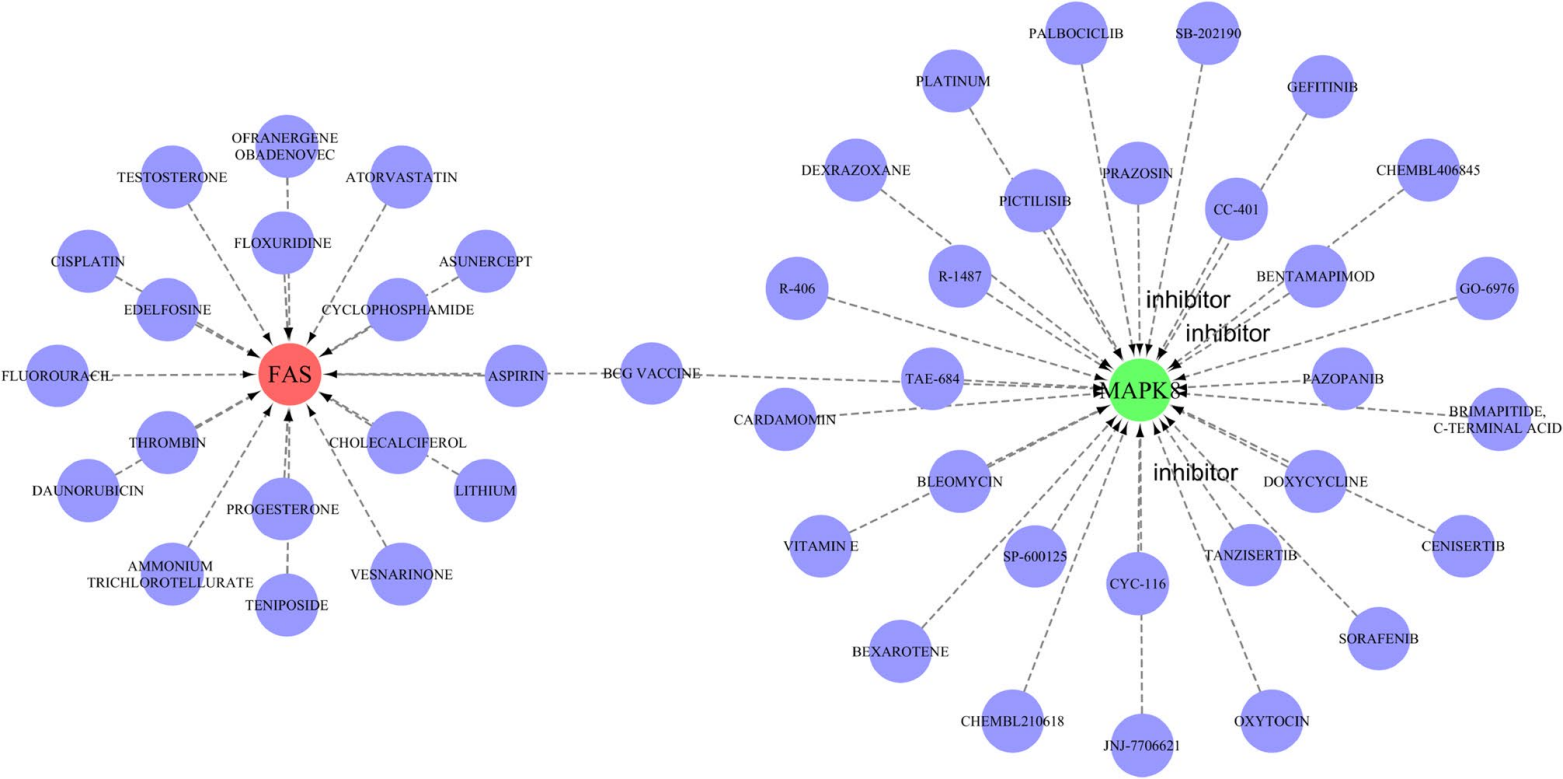
Prediction of marker gene-targeted drugs. Drug may target marker genes through DGIdb database and the interaction between the two.

**Figure 7:**
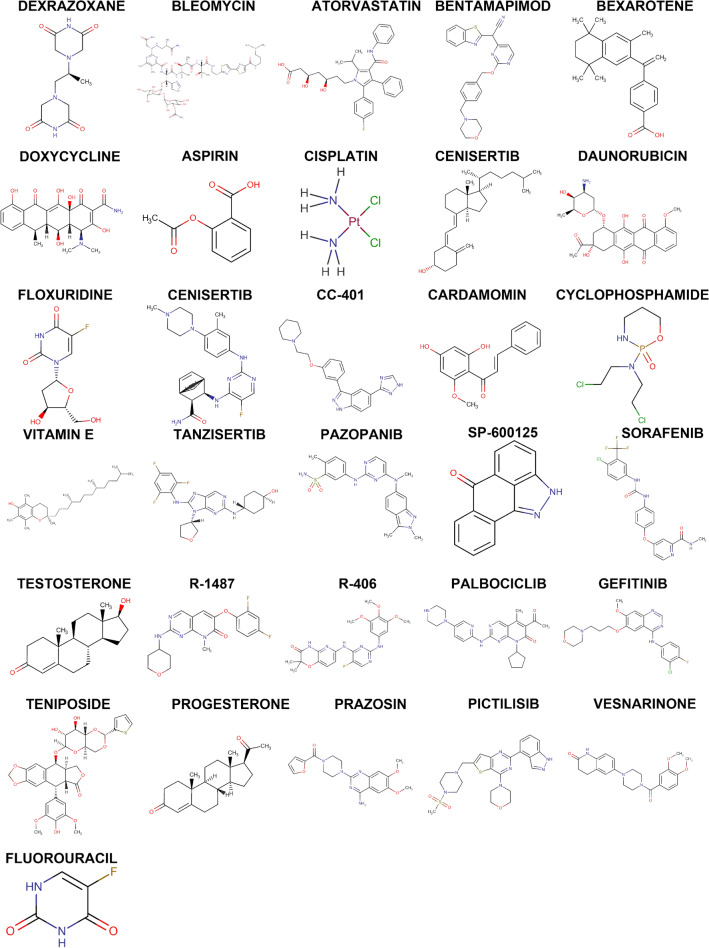
Chemical structure formula of the targeted drug.

**Figure 8:**
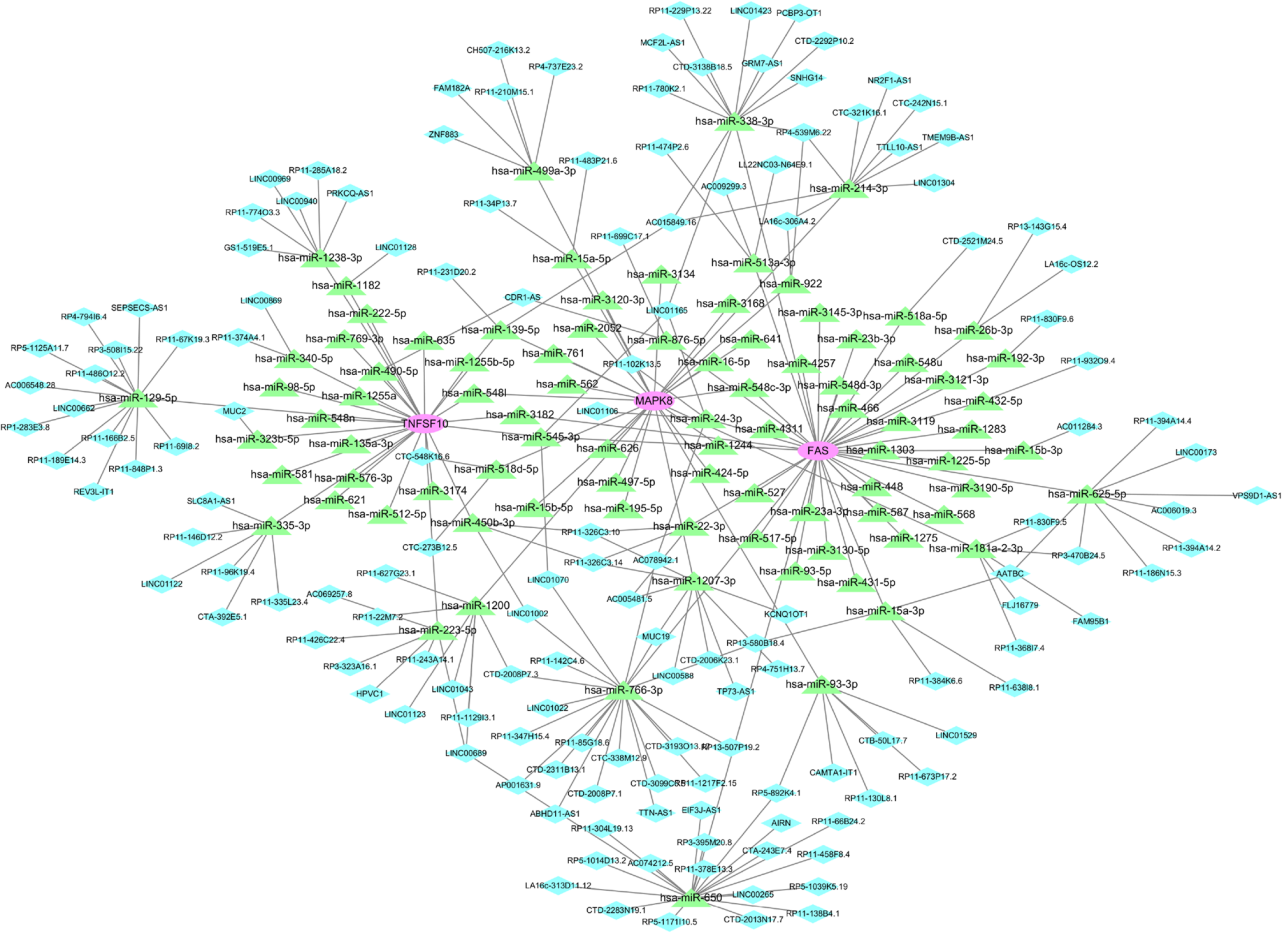
Key gene ceRNA network map. The network includes 238 nodes (3 key genes, 87 miRNAs and 148 lncRNAs) and 264 edges.

The DGIdb database led to the discovery of drugs that are likely to target the signature genes, and the Cytoscape software displayed the results in Fig. 6. There were 48 drugs targeting the signature genes, 19 of which targeted FAS, 39 targeting MAPK8, and no relevant targeting drugs were found for TNFSF10. Notably, among the predicted targeted drugs, TANZISERTIB, CC-401 and BENTAMAPIMOD are known inhibitors of MAPK8. In addition, we searched the structural formulae of the relevant target drugs through the DrugBank database, and as shown in Fig. 7, we retrieved a total of 31 chemical structural formulae among the predicted 48 target drugs. Our next step was to construct a ceRNA network by using the starBase and Miranda databases. As shown in Fig. 8, the network consisted of 238 nodes (3 key genes, 87 microRNAs, and 148 lncRNAs) and 264 edges. The number of lncRNAs that regulate FAS includes 59 lncRNAs that compete to bind hsa-miR-650, hsa-miR-625-5p, hsa-miR-766-3p, and hsa-miR-338-3p. in the ceRNA network, 20 lncRNAs can bind to hsa-miR-93-3p, hsa-miR-1238-3p and hsa-miR-214-3p to regulate the key gene MAPK8. hsa-miR-1238-3p and hsa-miR-214-3p to regulate the key gene MAPK8. Regarding TNFSF10, a total of 26 miRNAs were involved in regulation.

### Validation of expression of concentrated signature genes

**Figure 9:**
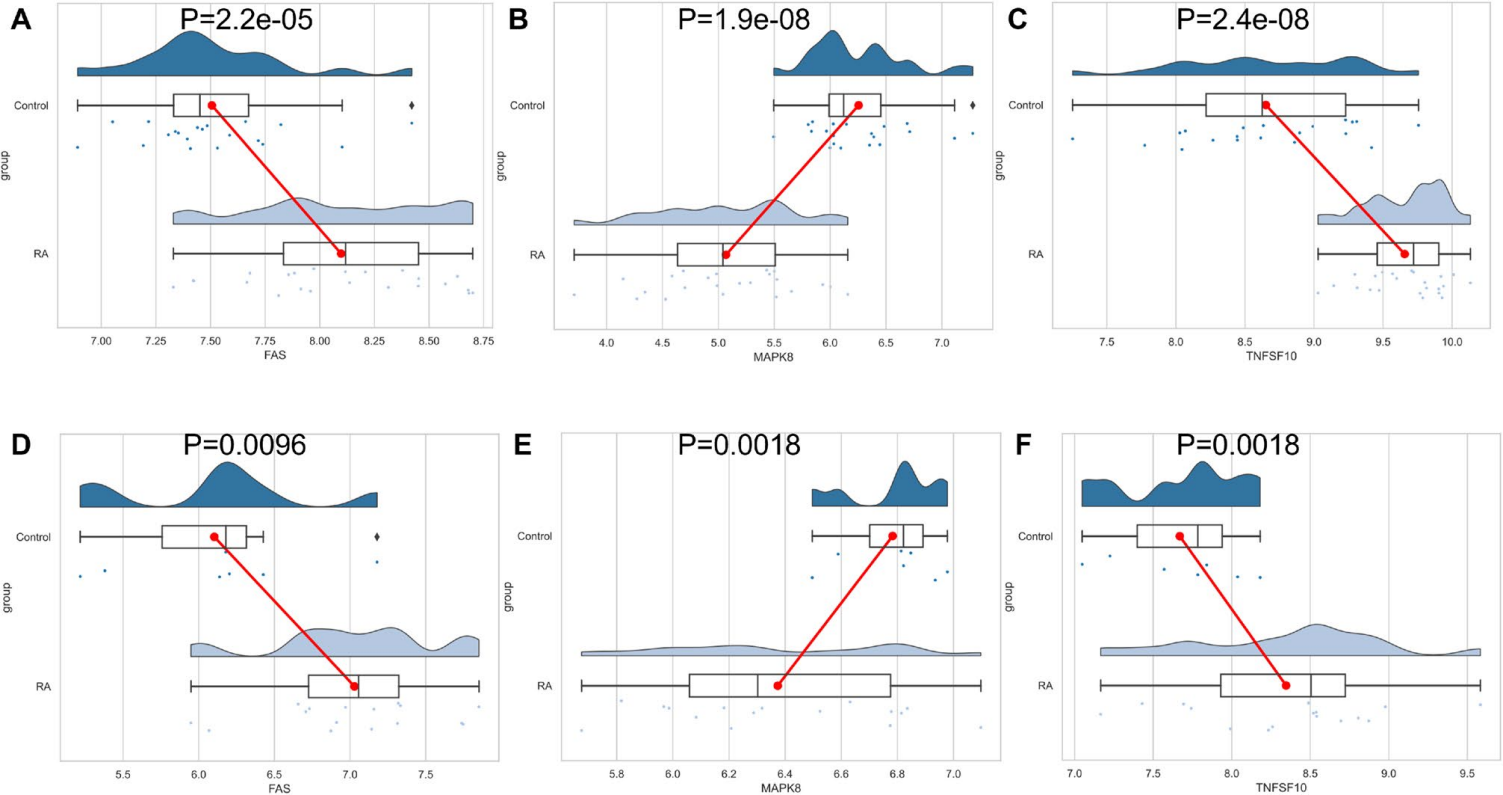
Key gene expression validation. (**A**–**C**) Expression of key genes in the training dataset. (**D**–**F**) Expression of key genes in the validation dataset.

The GSE77298 dataset was also used to validate the expression of key genes in RA samples. A comparison of the experimental data with the expression trends of three key genes (FAS, MAPK8 and TNFSF10) was found to be consistent. Among them, MAPK8 (*p* = 0.0018) was expressed lower in RA patients than in healthy control samples, while FAS (*p* = 0.0096) and TNFSF10 (*p* = 0.0018) were expressed higher in RA samples (Fig. 9).

## Discussion

Chronic inflammation and bone destruction of the synovium characterize RA^24^. An important cause of Rheumatoid Arthritis is abnormal proliferation of synovial fibroblasts and invasion into the synovial lining layer^25^. Since the pathogenesis of RA is complex and involves the regulation of several pathogenic genes, the analysis of RA differential genes and the improvement of the RA pathogenic gene network can provide directions for RA targeted therapy. There has been some research suggesting that necroptosis may play a role in the progression of RA, although it is not known how it works^26^. In this study, the RA synovial gene microarray dataset was selected to explore the molecular pathogenesis of Necroptosis in RA by seeking common features and analyzing potential associations between RA and Necroptosis-related genes through gene expression data analysis, enriched single-gene analysis, and enriched bioinformatics methods.

In this study, a total of three Necroptosis-related key genes (FAS, MAPK8 and TNFSF10) were obtained by three machine learning algorithms (LASSON, SVM-REF and RF) with the screening condition of gene importance greater than 0.9, etc. The AUC values represented by the area under the ROC curve of these three key genes in the training set were all greater than 0.85, and the AUC values in the validation set were all greater than 0.8, which can indicate that the key genes have certain accuracy and specificity for differentiating RA samples from healthy control samples. Apoptosis is triggered by FAS receptors on the surface of many types of cells and triggered by FAS ligand, the natural agonist to FAS receptors^27,28^. There has been a recent discovery of single nucleotide polymorphisms in the FAS and FASL promoters^29–31^. Compared to triple DMARD treatment, patients with FAS-670 GG genotype are more likely to have the genotype. Studies have shown that FAS-670's GG genotype can cause a decrease in FAS expression in synovial and T cells from patients with rheumatoid arthritis. Consequently, low FAS expression may interfere with the FAS-FASL pathway's apoptotic activity, resulting in greater synovial hyperplasia and inflammation^32^. Therefore, the increase in FAS in synovial tissues of RA patients may be caused by the activation of the FAS-FASL pathway leading to increased FAS expression or by further activation of the inflammatory response in vivo during synovial destruction in RA, which requires further investigation. Several studies have demonstrated that MAPK/ERK signaling regulates apoptosis and cell differentiation^33^. The Jun nuclear kinase (JNK), also known as MAPK8, has been shown to bind to and increase c-Jun transcription in response to radiation, environmental stress, and various growth factors^34^. In the synovial tissue of RA patients, abnormal activation of MAPKs promotes the formation of pannus formation^35^. Many studies have shown that TNFSF10 is a member of the tumor necrosis factor family. It can induce the death of cancer cells and inhibit the growth of tumors. Cancer cells^36^, for instance, die when they are induced to die by the p53 protein. There is evidence that TNFSF10 induces cytoprotective autophagy by activating the mitogen-activated protein kinase 8 pathway in lung, bladder, and prostate cancers^37^. Additionally, Huang^38^ et al. found that TNFSF10 stimulates proliferation and inflammation and inhibits apoptosis through miR-376-3p/FGFR1.The mechanism of TNFSF10 in RA has not been reported, but the present study found that TNFSF10 expression was inhibited. As well as controlling the progression of the disease, this may be a protective mechanism of the body. In addition to this, we found that the expression of receptor-interacting protein kinase 1 (RIPK1) was lower in the RA group than in the normal control group. RIPK1 is a protein kinase that regulates cell death and inflammation and is widely expressed in ovarian, lung, liver, intestine, and extremity tissues^39^. RIPK1 regulates apoptosis and necroptosis through both kinase-dependent and non-kinase-independent pathways, which are essential for cell fate and inflammatory response^40,41^. Raghav et al. found that in RA patients, compared to expression of TNF and its signaling intermediates, including RIPK1, has been shown to be constitutively increased in peripheral blood mononuclear cells compared to healthy controls^42^. Interestingly, the levels of RIPK1 and RIPK3 are reduced after controlling post-traumatic body temperature at 33 °C to reduce brain injury after stroke and TBI. These data suggest that deleterious RIPK1-dependent signaling may play a role in neuronal injury after TBI^43^. In summary, the high or low expression of RIPK1 may be closely related to the patient's test sample site and body temperature, in addition to the disease itself.

RA has a variety of pathogenetic mechanisms, and previous studies have only examined bone destruction in RA, but less from the perspective of immune inflammation, while immune dysfunction plays an important role in bone destruction in RA^44^. osteoporosis of RA joints is associated with T lymphocytes, B lymphocytes, helper T (helper T, TH) cells, and osteoblasts and T and B immune system cells interactions exist^45^. T cell CD8 and T cell CD4 memory activation were highly expressed in RA samples, while NK cell activation and mast cell activation were lower. It has been shown that T cells CD4 play a role in promoting pathological destruction of RA synovium and bone destruction^46,47^. CD4+ T cells include Th1, Th2, Th17 and Treg cells, and activated Th1 and Th17 cells are found in the RA synovial lumen during inflammation^48^. A number of studies have shown^49^ that NK cells play an important role in the pathogenesis of RA through intrinsic immunity. In addition to proliferating and activating NK cells, they cause the proliferation and activation of T and B lymphocytes by secreting cytokines, chemokines, or direct intercellular communication. Due to this, it contributes to the development and progression of synovial proliferation and bone destruction in RA. According to our analysis, these studies are valid.

Finally, we analyzed the targeting drugs and ceRNA networks of key genes. Among the forty MAPK8-targeting drugs retrieved, TANZISERTIB, CC-401, BENTAMAPIMOD, and HESPERADIN were shown to be inhibitors of MAPK8. As a predictive target of MAPK8, the drug HESPERADIN was shown to reduce arthritis scores, arthritis index, and inhibit serum levels of TNF-6, IL-17A, and CRP in CIA rats, and to exhibit analgesic, anti-inflammatory, and antipyretic potential in experimental animals^50^. A clearer understanding of the role MAPK8 plays in this process is still pending. In humans, non-protein-coding genes make up approximately 70% of the genome, and noncoding RNAs play an important role in autoimmunity and inflammation regulation^51^. An increasing number of studies have shown that some ncrna are specifically expressed in RA, mainly including microrna (mirna), long-stranded noncoding rna (lncrna) and circular rna (circrna)^52–54^. In order to determine whether our predicted key gene-targeting drugs and non-coding RNAs will work, specific pathways need to be explored. As a result, prospective studies of selected drugs and non-coding RNAs can be conducted.

In conclusion, we identified three key genes (FAS, MAPK8 and TNFSF10) associated with Necroptosis in RA samples. Aside from being closely associated with Necroptosis, these three genes are also associated with the immune microenvironment of synovial membranes in patients with RA. Although gene expression may not be directly equivalent to protein expression, and the biomarkers in this study should be considered as genes, not proteins, the significance of their study is undeniable. In order to better understand the pathogenesis and treatment of RA, we will continue to focus on these genes.

## Supplementary Information

Supplementary Information.

## Data Availability

The datasets and codes used during this study are available from the corresponding authors upon request.
